# Thrombolysis With Tenecteplase in Cardiac Arrest Due to Massive Pulmonary Embolism

**DOI:** 10.7759/cureus.93655

**Published:** 2025-10-01

**Authors:** Bhupinder Singh, Maninder Kansal, Ankita Soni, Rajiv Kumar, Suraj Kumar

**Affiliations:** 1 Cardiology, All India Institute of Medical Sciences, Bathinda, Bathinda, IND; 2 General Medicine, All India Institute of Medical Sciences, Bathinda, Bathinda, IND; 3 Pathology, All India Institute of Medical Sciences, Bathinda, Bathinda, IND; 4 Cardiothoracic Surgery, All India Institute of Medical Sciences, Bathinda, Bathinda, IND

**Keywords:** continuous renal replacement therapy (crrt), in-hospital cardiac arrest, in-hospital cpr, massive pulmonary embolism, systemic thrombolysis, tenecteplase (tnk)

## Abstract

Pulmonary embolism (PE) is a life-threatening condition that can cause sudden hemodynamic collapse. Prompt diagnosis and timely thrombolysis can be life-saving, even in situations where prolonged cardiopulmonary resuscitation (CPR) is necessary. We present a case of a morbidly obese patient who had a cardiac arrest due to massive PE. Immediate initiation of CPR and administration of tenecteplase resulted in the successful resuscitation of the patient after 45 minutes of CPR. With the help of supportive management and continuous renal replacement therapy (CRRT), the patient was successfully extubated on the third day and discharged home in a stable condition.

## Introduction

Pulmonary embolism (PE) is defined as the obstruction of the pulmonary arterial circulation by thrombotic material, most commonly arising from deep venous thrombosis of the lower extremities. The acute occlusion of pulmonary arteries results in a sharp rise in pulmonary vascular resistance, leading to right ventricular (RV) pressure overload, dilatation, and impaired systolic function. The failing RV reduces left ventricular filling, causing a fall in cardiac output, systemic hypotension, and ultimately cardiogenic shock. This cascade explains the rapid hemodynamic compromise seen in massive PE.

PE continues to be a major contributor to cardiovascular morbidity and mortality worldwide [1]. Its presentation can vary from mild symptoms to severe hemodynamic collapse. Massive PE, defined by severe hypotension, can quickly progress to cardiac arrest, especially in individuals who are obese or have pre-existing cardiac or pulmonary disease. While the prognosis of cardiac arrest due to PE is poor, favorable outcomes are achievable with prompt and aggressive intervention. Massive PE is associated with a 30% mortality, and it can reach 95% when PE causes cardiac arrest [2,3]. Several guidelines have supported the use of systemic thrombolysis during cardiopulmonary resuscitation (CPR) in the case of suspected or proven PE; however, clinical decision-making is still complex, particularly if a prolonged resuscitation attempt is involved. We present the case of a morbidly obese patient who suffered a sudden cardiac arrest in the emergency department.

## Case presentation

A 42-year-old morbidly obese (height 178 cm, weight 129 kg, BMI 40.7 kg/m²) male presented to our emergency department with an acute onset of dyspnea of six hours' duration. Earlier the same day, he had undergone a CT pulmonary angiogram at the radiology diagnostic center, which confirmed the presence of bilateral pulmonary emboli involving the main pulmonary arteries with extension into segmental branches. He was subsequently referred to our center by the general physician. No anticoagulation or thrombolysis was given at that time. The patient had a background history of type 2 diabetes mellitus and hypertension. There was no prior history of deep vein thrombosis or known hypercoagulable states.

**Figure 1:**
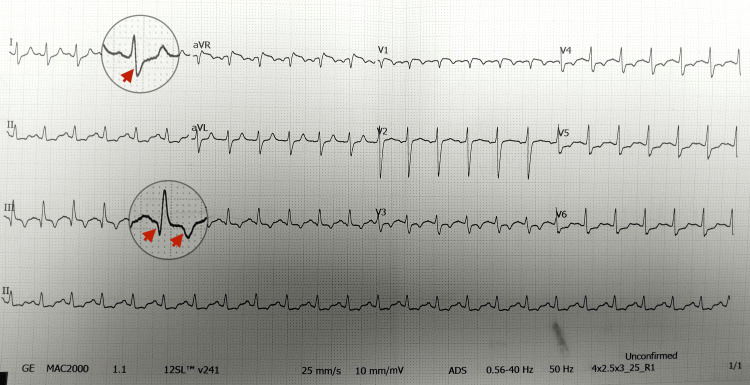
Baseline electrocardiogram on presentation The electrocardiogram (ECG) demonstrates sinus tachycardia with the classical S1Q3T3 pattern. Red arrows mark the prominent S wave in lead I, the Q wave in lead III, and the T wave inversion in lead III. These findings are characteristic of massive pulmonary embolism.

On arrival at our center, the patient appeared acutely ill and tachypneic. His vital signs revealed a heart rate of 130 bpm, blood pressure of 86/58 mmHg, respiratory rate of 30 breaths per minute, and SpO₂ of 85% on room air, improving to 94% with high-flow oxygen. The physical examination revealed jugular venous distension and clear lung fields. The initial 12-lead electrocardiogram (ECG) demonstrated sinus tachycardia with an S1Q3T3 pattern and T wave inversions in V1-V4, suggesting RV strain (Figure 1).

Given the recent CT findings and hemodynamic instability, a bedside transthoracic echocardiogram (TTE) was immediately performed (Video 1).

**Video 1:** Transthoracic echocardiography at presentation Transthoracic echocardiography (TTE) demonstrates severe right ventricular (RV) dilatation with interventricular septal flattening, producing a "D-shaped" left ventricle. Severe tricuspid regurgitation and pulmonary artery hypertension are noted. McConnell's sign (akinesis of the mid-RV free wall with preserved apical contractility) is present. Progressive bradycardia is also observed, heralding imminent cardiac arrest.

It revealed RV dilation with severely reduced RV systolic function, McConnell's sign (akinesis of the mid-free wall with preserved apical contractility), and severe pulmonary artery hypertension with pulmonary artery systolic pressures (PASP) of 70 mmHg. During the baseline evaluation, midway through the scan, the patient became unresponsive and pulseless. The cardiac monitor confirmed pulseless electrical activity (PEA).

Advanced cardiac life support (ACLS) protocols were initiated without delay. High-quality CPR was provided continuously with mechanical ventilation support. Intravenous access was secured, and adrenaline was administered every three to five minutes in recommended doses. Given the recent CT findings and echocardiographic evidence suggesting massive PE as the cause of cardiac arrest, and in the absence of contraindications, a 50 mg IV bolus (weight-based) of tenecteplase was immediately administered. Alteplase was not used because tenecteplase allows single-bolus dosing and has high fibrin specificity, making it more practical in the CPR setting [4]. The extracorporeal membrane oxygenation (ECMO) protocol was activated early; however, extracorporeal support could not be initiated as the patient's family declined consent due to financial constraints.

CPR was continued for approximately 45 minutes. The patient eventually achieved a return of spontaneous circulation (ROSC) with a palpable pulse, detectable blood pressure, and sinus rhythm on the ECG. An arterial blood gas (ABG) obtained post-ROSC revealed severe metabolic acidosis with a pH of 6.9, bicarbonate of 8 mmol/L, and a lactate level of >15 mmol/L. He was shifted to the intensive care unit (ICU) for post-resuscitation care.

In the ICU, the patient was mechanically ventilated. Initial ventilator settings were volume control mode, tidal volume 6 ml/kg predicted body weight, FiO₂ 100%, and PEEP 8 cm H₂O. These settings were gradually reduced, and he was successfully extubated on day three. Norepinephrine infusion was used as the main vasopressor to maintain perfusion. Laboratory investigations revealed acute kidney injury with only a modest rise in creatinine (from 1.1 to 1.6 mg/dL), but the patient developed anuria. Continuous renal replacement therapy (CRRT) was initiated to correct metabolic acidosis and maintain fluid balance. The serial laboratory investigations highlighting the metabolic abnormalities are presented in Table 1.

**Table 1:** Serial laboratory investigations This table summarizes arterial blood gas (ABG) and renal function parameters at different time points.

Parameter	During CPR	Post-ROSC	6 Hours Post-ROSC	12 Hours Post-ROSC	Day 3 (Pre-extubation)	Day 4
pH	6.8	6.9	6.9	7.25	7.34	7.4
Bicarbonate (mmol/L)	8	8	10	12	20	24
Lactate (mmol/L)	>15	>15	>10	6	3	1.5
Creatinine (mg/dL)	-	-	1.6	1.8	1.2	1.2
Urine output	Anuric	Anuric	Anuric --> CRRT started and continued for 12 hours	CRRT Continued	Normal	Normal

**Figure 2:**
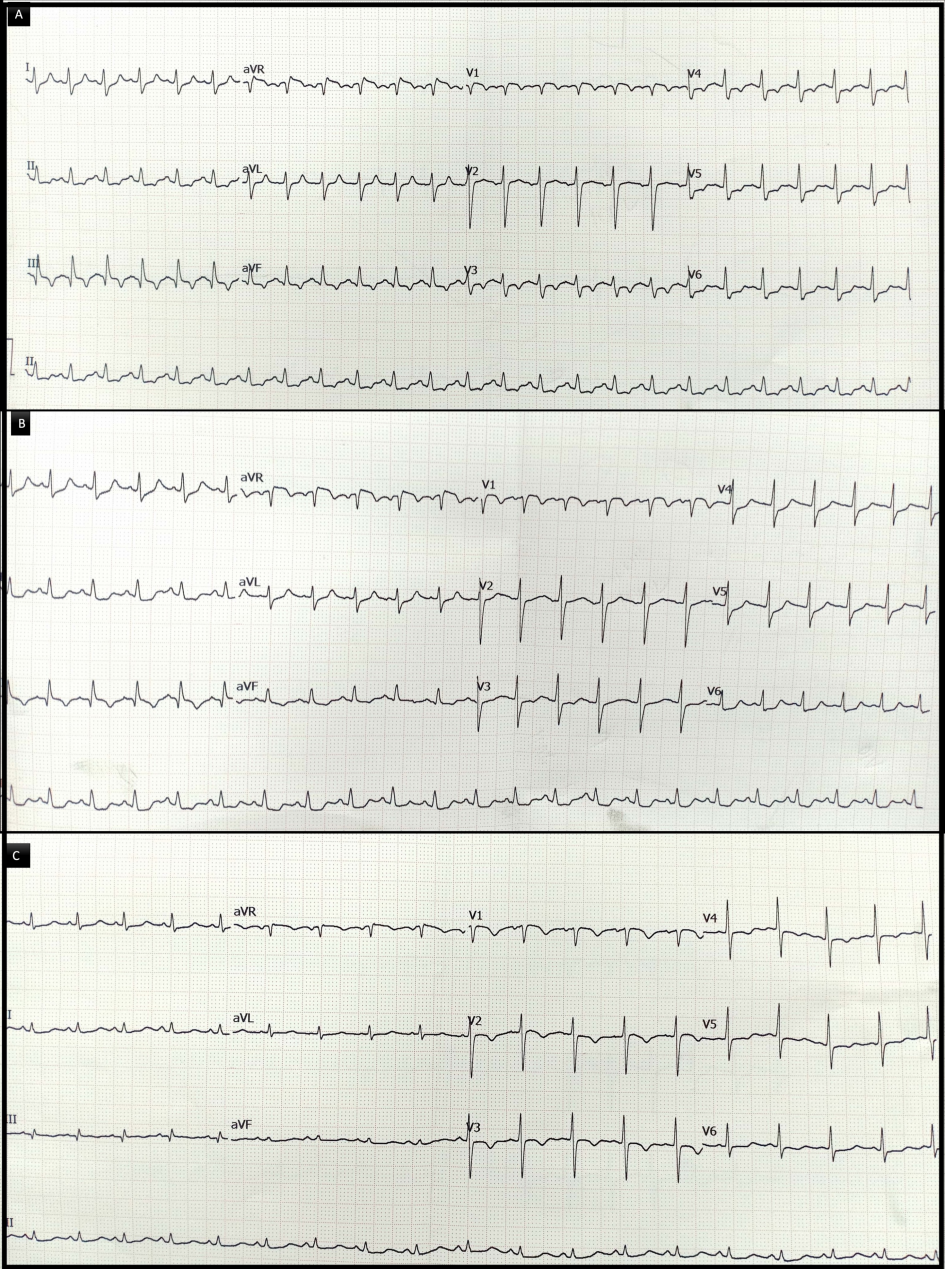
Evolution of electrocardiographic changes after thrombolysis Serial electrocardiograms (ECGs) over 24 hours following thrombolysis. (A) Three hours post-return of spontaneous circulation (ROSC): sinus tachycardia with persistent S1Q3T3 pattern. (B) Twelve hours post-ROSC: persistence of S1Q3T3 with slight resolution of ST-T wave abnormalities. (C) Twenty-four hours post-ROSC: resolution of the S1Q3T3 pattern with near-complete resolution of ST-T abnormalities.

Serial ECGs and bedside TTEs were performed at 3, 12, and 24 hours post-ROSC to monitor recovery and RV function (Table 2, Figure 2).

**Table 2:** Serial electrocardiogram and echocardiographic findings following thrombolysis and prolonged resuscitation This table summarizes the patient's electrocardiogram (ECG) and transthoracic echocardiographic (TTE) findings at 3, 12, and 24 hours post-return of spontaneous circulation (ROSC).

Time Post-ROSC	Electrocardiographic Findings	Echocardiographic Findings
3 hours	Sinus tachycardia persists; classical S1Q3T3 pattern; ST-T changes consistent with right heart strain.	Persistent right ventricular (RV) dilation and dysfunction, but a reduction in pulmonary artery systolic pressure (PASP) to 50 mm Hg.
12 hours	Persistence of the S1Q3T3 pattern, and slight resolution of ST-T wave abnormalities.	Reduction in RV size with slight improvement in RV function; PASP reduced to 35 mm Hg.
24 hours	Resolution of the S1Q3T3 pattern; ST-T wave abnormalities also significantly resolved.	Further reduction in RV size and improvement in RV systolic function; PASP reduced to 26 mm Hg.

Anticoagulation was continued with low-molecular-weight heparin, which was subsequently transitioned to oral apixaban. Compression ultrasonography of the lower limbs revealed no evidence of deep vein thrombosis. With continued clinical improvement, the patient was discharged home on day 10, exhibiting stable vital signs, normal renal function, and near-complete recovery of cardiac function on echocardiography.

## Discussion

This case highlights the critical importance of early recognition and aggressive management of massive PE, particularly in patients with high-risk profiles such as morbid obesity. PE is one of the major differential diagnoses in patients presenting in the emergency department with an acute onset of dyspnea and hypotension. In these situations, a bedside echocardiogram plays a crucial role, as it provides vital information before laboratory or radiologic confirmation.

According to a meta-analysis of patients who suffered cardiac arrest as a result of a PE, it was shown that fibrinolytic therapy was associated with improved rates of ROSC, survival until hospital discharge, and also favorable long-term neurological outcomes [5]. Current guidelines from the European Resuscitation Council (ERC) and the American Heart Association (AHA) also support the use of systemic thrombolysis during CPR for PE [6,7]. Patients suffering from cardiac arrest after PE also benefit from ECMO, surgical embolectomy, and percutaneous mechanical thrombectomy in addition to systemic thrombolysis [6-8]. The ECMO protocol was activated at the time of cardiac arrest, but the patient's family did not provide consent. Surgical thrombectomy remains a therapeutic option for massive PE, but it is not practical once CPR has already been initiated.

When PE is known or highly suspected as the cause of cardiac arrest, prolonged CPR (of at least 60-90 minutes) with continued resuscitation efforts is recommended. Wu and colleagues reported a good outcome after thrombolysis in a patient who underwent 100 minutes of CPR [9]. Renkes-Hegendörfer and Hermann were among the first to describe a successful outcome after administering streptokinase during cardiac arrest due to PE [10]. Subsequently, effective use of other thrombolytic agents, such as urokinase, recombinant tissue plasminogen activator (rt-PA or alteplase), reteplase, and tenecteplase, has been reported [11-14]. Tenecteplase, a genetically altered version of alteplase that facilitates bolus delivery and increases fibrin specificity, was selected for our patient because it is simple to utilize during active cardiopulmonary resuscitation. Its pharmacologic advantages and single-bolus delivery made it particularly suitable in the chaotic context of cardiac arrest management.

The favorable neurological recovery in our patient may also be partially explained by the proposed neuroprotective effects of thrombolytic therapy, although the exact mechanism is not fully understood [15]. Some authors suggest that thrombolytics may attenuate the secondary thrombotic cascade initiated by cardiac arrest, reducing microvascular occlusion and inflammatory injury [16,17]. Additionally, thrombolysis may lower the incidence of arrhythmias and improve overall myocardial perfusion following PE-related arrest [18].

Furthermore, it is hypothesized that chest compressions in a patient with an acute PE cause mechanical fragmentation of the thrombus while augmenting microcirculation reperfusion. This mechanical effect may facilitate more effective thrombolysis and help restore pulmonary perfusion during CPR [19].

Prolonged CPR, lasting approximately 45 minutes in this case, is generally associated with poor outcomes, particularly regarding neurological function. However, when the etiology is reversible, such as thrombotic PE, and when CPR quality is high with minimal interruptions, survival with good neurological outcomes becomes achievable. The favorable outcome in our patient emphasizes the importance of perseverance and early targeted intervention during resuscitative efforts.

In addition to thrombolysis, supportive care in the ICU played a vital role in recovery. CRRT played a pivotal role in addressing post-resuscitation complications, including acute kidney injury and metabolic acidosis. Timely renal support, especially in critically ill obese patients, can facilitate hemodynamic stability and overall recovery by managing fluid overload and clearing metabolic toxins.

Serial ECGs and bedside echocardiography were pivotal in tracking the patient's cardiopulmonary recovery after thrombolysis and prolonged resuscitation. Initial findings of sinus tachycardia, an S1Q3T3 pattern on the ECG, and severe RV dilatation and dysfunction supported the diagnosis of massive PE. Gradual normalization of RV size and function over 24 hours, as well as resolution of ECG abnormalities, reflected successful reperfusion and RV unloading. In addition to offering reassurance regarding hemodynamic stability, these serial evaluations led to modifications in supportive care, particularly the de-escalation of ventilatory support and vasopressors.

It is crucial to remember that the patient had CT imaging done prior to arrival in the emergency department and had already been diagnosed with PE. This made it easier to make quick decisions and perform thrombolysis in the event of cardiac arrest. Point-of-care echocardiography is even more crucial in situations where the diagnosis is unclear.

This case emphasizes how important it is to keep PE as a differential in cases of undifferentiated shock or arrest, particularly in patients who have risk factors including obesity, immobility, or recent surgical intervention. It also demonstrates the effectiveness of integrated multidisciplinary care, with emergency physicians, cardiologists, nephrologists, and intensivists working together to reach a favorable result.

## Conclusions

Massive PE should always be suspected in sudden, unexplained dyspnea with hypotension and collapse. Quick use of bedside echocardiography can guide diagnosis. In cardiac arrest due to PE, thrombolysis should be considered immediately, even during ongoing CPR. Tenecteplase is a simple and effective choice due to the bolus dose. Prolonged high-quality CPR should be continued if the cause is potentially reversible. Multidisciplinary ICU care, including ventilator, vasopressor, and CRRT support, also plays a major role.

This case further highlights that the delay in initiating therapy occurred because the patient was referred from a diagnostic center after CTPA without anticoagulation or thrombolysis. Despite this, the timely administration of tenecteplase during CPR at our center led to ROSC and survival with a good neurological outcome. It underscores the importance of early initiation of therapy and persistence during resuscitation.
